# Level of completion along continuum of care for maternal and newborn health services and factors associated with it among women in Arba Minch Zuria woreda, Gamo zone, Southern Ethiopia: A community based cross-sectional study

**DOI:** 10.1371/journal.pone.0221670

**Published:** 2020-06-08

**Authors:** Dereje Haile, Mekdes Kondale, Eshetu Andarge, Abayneh Tunje, Teshale Fikadu, Nigussie Boti

**Affiliations:** 1 Wolaita Zone Health Department, Duguna Fango Health office, Wolaita Sodo, Wolaita Zone, Ethiopia; 2 Reproductive Health Unit, School of Public Health, College of Medicine and Health Sciences, Arba Minch University, Arba Minch, Ethiopia; 3 Epidemiology and Biostatistics Unit, School of Public Health, College of Medicine and Health Sciences, Arba Minch University, Arba Minch, Ethiopia; University of Tokyo, JAPAN

## Abstract

**Background:**

Completion along continuum of care for maternal and newborn health (MNH) services like antenatal care, skilled birth attendance and postnatal care services is advantageous over each segment of services. It is one of the currently recommended strategies to reduce both maternal and neonatal mortality and achieve the global target of ending preventable maternal and under-five children’s mortality. Although studies on factors affecting each segment of MNH services have been well documented in Ethiopia, there is a dearth of evidence about the level of continuum of care and factors associated with it. This study was intended to fill this gap in evidence in the study area so that interventions could be taken to improve maternal and newborn health.

**Methods:**

A community-basedcross-sectional study was conducted among 432 postnatal women who gave birth in the previous year in Arba Minch Health and Demographic Surveillance System (HDSS) site. Women were selected by computer generated random numbers from a list of women who stayed at least 6 weeks after birth. A pre-tested, structured, and interviewer-administered questionnaire was used for data collection. Data were entered and coded in Epi-data and analyzed using SPSS software version 23. Binary logistic regression model was fitted to identify factors associated with the dependent variable. Bivariate and multivariable analyses were fitted in steps to select candidate variables for multivariable analysis and to control for potential confounding effect respectively.

**Results:**

The overall completion along the continuum of care was 42(9.7%). The factors significantly associated with completion of care along the continuumwere timely initiation of antenatalcare (before16weeks) [AOR: 10.7, CI (5.1, 22.7], birth preparedness and complication readiness [AOR: 2.9, CI (1.4, 6.1), pre-pregnancy contraception utilization [AOR: 3.9, CI: 1.4, 11.0], being employed [AOR: 2.6 CI:(1.3, 5.4)], and having a planned pregnancy [AOR:3.5 CI: (1.1, 11.4)].

**Conclusion and recommendation:**

Completion along the continuum of care for MNH services was low in the study area. Thus, efforts to improve the completion of care should focus on interventions that enhance early initiation of antenatal care, planned pregnancy, and birth preparedness and complication readiness.

## Introduction

Maternal morbidity and mortality is a major public health problem of the globe even thougha high discrepancy exists between high and low income countries [1, 2]. According to the World Health Organization(WHO) report in 2015, 99% of maternal deaths in the world occurred in low andmiddle-income countries(LMICs). This occurs in a largest toll of an estimated 66% of the cases in the Sub-Saharan Africa region [1]. The maternal mortality ratio (MMR) was about 20 times higher inthe LMICs when compared with themortality in highincomecountries. Likewise, the life time risk of maternal death was also about 27 times higherinthe LMICs. Despite the progress in coverage of maternal, newborn, and child healthservices in the last 25 years, a high MMR(412 maternal deaths per 100,000 live births) was reported in the 2016 Ethiopian Demographic Health Survey(EDHS) [3].

Even though high MMR is a problem of concern, nearly 75% of all maternal deaths are duetodirectobstetriccausesandarepreventablewithextremelycost-effective interventions [4]. The Sub-Saharan Africa and South East Asia regions account for 80% of the total neonatal deaths worldwide. A child born in these regions is nine times more likely to die in the first month of life than a child born in a high-income country [5]. According to the 2017 United Nations Children's Fund(UNICEF) report, Ethiopia was categorized with India, Pakistan, Nigeria, the Democratic Republic of the Congo as accounting for half of world-wide newborn deaths [6].

Continuum of care (CoC)which usually refers to continuity of the individual MNH caresis one of the health services strategic frameworks and indicators of maternal, newborn, child and adolescents’ health. It is currently given more attention and recommended as advantageousovereach care provided in separate [7]. It has two dimensions: time and place. CoC at its time dimension refers to a situation where a woman and her child receive MNCH services along the continuum of pre-pregnancy, pregnancy, childbirth, to postpartum and newborn through childhood respectively. The place dimension focuses on integration among household level, community-level and facility-level MNH cares as well as referral to advanced-level care when needed [8, 9].

Completion along CoC for MNHservices such as antenatal care(ANC), skilled birth attendance and postnatal care(PNC) services is one of the strategies to reduce both maternal and neonatal mortality [9]. Furthermore, improvement in CoC for MNH services or life -course approach was one of the recommendations to achieve the global target of reduction of maternal mortality (70 maternal deaths per 100,000 live birth) and ending preventable under-five child mortality [10]. Complete exposure to theCoC has advantages over separate cares or interventionsbecauseeach stage of the CoC builds on the success of the previous stages [11–16]. However, evidence from the globe suggest that women completing the CoC for MNH services are very low and women do not access MNH services alongthe CoC [17–19].

The Federal Ministry of Health of Ethiopia has implemented many high impact interventions in recent years to improve MNH services and the country has made good progress in reducing MMR [20]. However, a remarkably low progress has been made in each segment of the MNH servicesaccording to the successive EDHS reports [3]. The level of completion along the three continuums ANC, skilled birth attendance and PNCis also one of the lowest in comparison with other sub-Saharan African countries [21, 22].

Existing studies in the country focused on the first level (ANC completion) and second level continuums (ANC to skilled birth attendance) [23–26]. Studies considering the completion and dropouts in the postnatal continuum were missing while this being the most underutilized service [27, 28] as well as a critical period to save lives of women and newborns [28–30]. To the best of our knowledge, there was a recently published study in the country based on secondary data from EDHS2016 which reported low completion(9.1%) along the three continuums of services [22]. Although the study brought about an important evidence from a large nation-wide sample of women and employed an advanced statistical analysis, the definition of incomplete continuum might have overestimated the overall level of CoC as it considered women who received ANC4+ and skilled birth attendance by leaving out those women who failed to complete the four ANC and who did not receive skilled delivery care. Even though this study was limited in a small locality in Southern Ethiopia, it has considered women who failed to complete the four ANC and who did not receive skilled delivery care as women having incomplete continuum.

In response to the limited evidencein the country in recent yearsand to fill this gap in the scientific literature, our study aimed to assess the level of CoC for MNH services and identify factors associated with it among women in Arba Minch Zuria woreda HDSS site, Southern Ethiopia. Given the high toll of maternal and newborn morbidity and mortality in the country, the findings of this study could assist in programme planning and policymaking.

## Methods

### Study setting and design

The study was conducted in Arba Minch Zuria Woreda, Gamo zone, Southern Ethiopia, at 454 km Southof Addis Ababa, the capital city of Ethiopia. The woreda has a total of 29 kebeles; two of which are semi-urban and the remaining 27 are rural. *A woreda is an administrative unit corresponding to district in other parts of the world and a kebele is the smallest administrative unit in the current Ethiopian government structure under woredas*. The Arba Minch HDSS site works within nine kebeles (one semi-urban, eight rural) in the woreda which were selected randomly after stratifying the woreda in climatic zones in to high land and low land areas. The surveillance site has permanently recruited data collectors who use the Open Data Kit (ODK) mobile phone- based application for data collection and report monthly to the center located at Arba Minch University. In the HDSS site, there was continuous registration and recording of data on maternal and child health services. The data consisted of different health and health related informationof women in relation to their pregnancy, labor and delivery, PNC as well as nutritional status. A community-based cross- sectional study was conducted from 15 February to 15 March 2019.

### Population of the study

The source population for this study were all women in Arba Minch Zuria Woreda who started ANC in the primary public health facilities (health posts and health centers) and stayed six or more weeks after giving birth. The study population were women in the nine kebeles of Arba Minch HDSS site who started ANC at health facilities and stayed six or more weeks after giving birth. Women who lived in the study area for less than six months and who were critically ill at the time of data collection were excluded from the study.

### Sample size determination

The sample size for this study was initially calculated using statcalc menu of Epi-info software version 7 [31] using the assumptions for single population proportion with estimated prevalence of 50% (because of the absence of studies in a similar setting), 95% confidence and 5% degree of precision. The sample size calculated using these assumptions was 384. Then, assumptions for a two-population proportionwere made with consideration of factors affecting CoC for MNH care. Among the biological plausible factors selected, the largest sample size(n = 398) was obtained by considering 22.9% CoC among women who had five or more children, AOR of 1.91 [32], 80% power, 95% confidence level, 5% degree of precision, and the ratio of unexposed to exposed is equal to 1. Since the sample size obtained using two population proportion considerations (n = 398) was larger than the sample size for single population proportion (n = 384), it was used as the sample size for the study. An additional 10% was considered to compensate for potential non-response, and the final sample size for this study was 438.

### Sampling technique

To obtain the sampling frame, a secondary data was used from the HDSS site data base. First, a total of 1,316 women who gave live birth the previous year(December 2017 to December 2018) prior to our survey period were registered in the HDSS site data base. Second, a total of 595 women who started ANC in the primary health care facilities (health centers and health posts) and stayed 6 or more weeks after child birth were selected out of the initial 1,316 women. A sampling frame of these 595 women was used to select the sample for this study. The required number of women for the study (n = 438) were selected after proportional allocation of women to each kebele based on the number in the respective kebeles. Thus, a separate list of women was prepared for each kebele and the required samples were selected using computer generated random numbers (S1 Fig).

### Data collection methods and procedures

Nine experienced data collectors of the HDSS site collected the data through a face-to-face interviewand three public health officersworking in the district supervised the data collection. Both data collectors and supervisors were given a one-day intensive training on the data collection methods and instruments. The socio-demographic and obstetric data were collected from women through semi-structured interviewer- administered questionnaires, andhousehold socio-economic status was collected using astructured questionnaire. The questionnaire was adapted from the EDHS 2016 questionnaire for women and household characteristics, and related published literatures with consideration of constructs from Anderson’s model on health care-seeking [3, 11, 17, 18, 32–35]. The supervisors and data collectors accessed houses of the sampled women by the guidance of the local health development army leaders in each kebele. The list of women selected for the interview in their respective kebeles was provided tothe data collectors in advance.

### Data quality management

Intensive training was provided to the data collection team by the team of investigators with particular focus on the contents of the questionnaire. A practical role play on interviewing skills was exchanged among the data collection team. The questionnaire used to obtain data from the women was initially prepared in English and translated to the local language by an expert in the language and finally back translated to English by another expert to check its consistency with the original meanings. The final questionnaire in the local language was used for data collection. Before commencing data collection, pre-testing was conducted in a similar setting outside the study area at Chencha woreda on 5% of the sample (22 women). In response to the pre-test findings, some amendments were made to alter unclear and confusing questions. To minimize social desirability bias, women were interviewed in a separate private place in their own household compound.

### Data analysis

Data were entered in to Epi-data software version 3.1 and then exported to SPSS version 23 for analysis [36]. Descriptive statistics were used to quantify level of CoC for MNH services and other explanatory variables. Findings were summarized in tables and graphs using frequencies, percentages and standard deviations. The household wealth status of the study womenwas constructed using principal components analysis (PCA). Initially, 39 items composed of householdproductive and non-productive assets as well as utilities were entered for the analysis. If a variable/asset was owned by more than 95% or less than 5% of the sample, it was excluded from the analysis because it would not help to distinguish between richer and poorer households. After the first exploration the variables using frequencies, satisfaction of assumptions for PCA was assessed by the Kaiser-Meyer-Olkin measure of sampling adequacy (≥ 0.6) and Bartlett‘s test of sphericity(p-value < 0.05). In each step, variables with anti-image correlations and communalities less than 0.5, having a loading (correlations higher than 0.4) in more than one component (having complex structure), and a single variable loading in a component were removed until the iterations fulfilled the criteria. Finally, three components which explained a total variance of 69.7% were extracted from the PCA. Among these, the first component explained the largest variance of 33.6%. The items remained in the first component were ownership of farm land and domestic animals (milk cow/cow/bull), and size of land. A factor score of this component was used to rank household wealth status of the study women into quintiles.

Binary logistic regression model was fitted to identify factors associated with CoC. Model fitness was checked by the Hosmer and Lemeshow goodness of fitness test and multi-collinearity between the explanatory variables was checked using the variance inflation factor (VIF>10). Initially, bivariate logistic regression analysis was performed between dependent and each of the independent variables in sequence. Variables having a p-value<0.25 in bivariate logistic regression were selected as candidates for multivariable logistic regression analysis and were entered sequentially by using backward stepwise regression. Association between outcome variable and independent variables was reported by using adjusted odds ratioandits95% CI, andvariables having a p-value ≤ 0.05 in multivariable logistic regression model were regarded as statistically significant.

### Definition and operationalization of variables

The dependent variable for this study was CoC for MNH services. The completion status was classified as ‘completed the continuum’ or ‘not completed the CoC. A woman was regarded as completed the continuum when they hadreceived the three MNH services along continuum from a skilled provider. These include four or more ANC visits, childbirth at a health facility, and PNC within 6 weeks after childbirth(at least once after discharge from health facilities or within the first week after childbirth at theirhome). She was classified as not completed the CoC if she missed any one of the above visits/attendances at any level [17, 32, 37, 38].

Skilled provider:A health care professional such as doctor, nurse, midwife, health officer or health extension worker working in the health facilities and who has got the necessary pre-service and/or in-service training for the provision of MNH services [3].

Autonomy in household decision making: A woman was said to have autonomousdecision making powerin seeking MNH services if she alone or with her husband (jointly) decidedon seeking MNH services; otherwise (if her husband alone or a third person decided on seeking MNH services) she was considered as not having autonomous decision making power [18].

Knowledge on key pregnancy danger signs: A woman was classified as knowledgeable if shespontaneously mentioned at least two of the four key danger signs of pregnancy (vaginal bleeding, severe headache, blurring of vision and swelling of feet or face); if not she was classified as not knowledgeable [17].

A woman was considered as ‘well prepared’ for birth and its complications when shereported that she has implemented five or more components of birth preparedness and complication readiness (BPCR); otherwise she was considered as ‘not well prepared’. The components of BPCR considered in this study were identified place for birth, identified skilled birth attendants, saved money, identified transportation for emergency conditions, identified a companion duringlabor and birth, identified blood donors, and identified care giver to children at home when the mother was away [39, 40].

Signs of postpartum complications: major signs that indicated occurrence of complications after expulsion of fetus within 42 days such as severe vaginal bleeding, fever, urinary incontinence, and/or offensive/foul smelling vaginal discharge [41].

Immediate postpartum contraception utilization: Women’s use of any type of modern methods of contraception in the postpartum period before discharge from the health facility [42].

### Ethics approval and consent to participate

Ethics clearance was obtained from the institutional research ethics review board of Arba Minch University. Respondents were informed about the purpose and procedures of the study. Oral consent was obtained from the study women since about half of them did not attend a formal education and were not able to give a written consent. A unique ID number was assigned to each questionnaire in order to make it anonymous. Privacy and confidentiality of information of the women was assured before obtaining data. With regard to confidentiality, respondents were given information that guaranteed them that the information they provided during the study would be used for the research purpose and would not be disclosed to anybody outside the research team. Aformal letter of permission was obtained from Gamo zone health department and Arba Minch zuria woreda health office.

## Results

In this study, 432 women volunteered to participate, yielding a response rate of 98%.

### Socio-demographic characteristics of the study women

The median age of the respondents was 27.5 years (IQR = 5), and the majority (96.2%) were married. More than a third (40.1%) of themwere unable to read and write, and more than half (61.8%) were unemployed (Table 1).

**Table 1:** Socio-demographic characteristics of the study women in Arba Minch HDSS site, February 15-March 15, 2019.

Respondents’ characteristics	Category	Frequency	Percentage
**Age (n = 432)**	18–24	93	21.5
	25–29	197	45.6
	30–34	88	20.3
	> = 35	54	12.5
**Marital status (n = 432)**	Single/divorced/widowed	16	3.8
	Married	416	96.2
**Religion (n = 432)**	Orthodox	131	30.3
	Protestants	298	69.7
	Others	3	0.7
**Educational status(n = 432)**	Unable to read or write	173	40.1
	Able to read or write	51	11.8
	Primary level of education	155	35.8
	Secondary and above	53	12.3
**Employment status(n = 432)**	Unemployed	267	61.8
	Employed	165	38.2
**Husband’s educational status(n = 412)**	Unable to read or write	104	25.3
	Able to read or write	58	14.1
	Primary level of education	180	43.7
	Secondary education and above	70	16.9
	Lowest	84	19.4
**Household wealth status**	Second	85	19.7
	Middle	91	21.1
	Fourth	86	19.9
	Highest	86	19.9

### Women health service access and behavior related characteristics

Regarding accessibility of MNH care services, more than half (58.3%) reported that the average time to reach health facilities was less than thirty minutes. Majority of the study women (79.6%) made decisions for maternity care autonomously (Table 2).

**Table 2:** Women’s access to service and behavior related characteristics among the study women in Arba Minch HDSS site, February 15-March 15, 2019.

Respondents’ characteristics	Category	Frequency	Percentage
**Means of transportation to health facilities(n = 432)**	By motor cycle/car	73	16.9
	On foot	359	83.1
**Time spent to reach health facilities(n = 432)**	< 30 minutes	252	58.3
	> = 30 minutes	180	41.7
**Exposure to mass media (radio/television) (n = 432)**	Yes	240	55.6
	No	192	44.4
**Frequency of exposure to mass media(n = 240)**	Always	65	27.1
	More than once a week	60	25
	Once a week	115	47.9
**Membership of community-based health insurance (n = 432)**	Yes	222	51.4
	No	210	48.6
**Autonomy in decision-making powerto maternity care (n = 432)**	Autonomous	344	79.6
	Not autonomous	88	20.4

### Obstetric characteristics of the study women

Regarding the obstetric history of respondents, the majority (73%) were multiparous. About two-third (60.4%) of the study women were not knowledgeable about pregnancy danger signs (Table 3).

**Table 3:** Obstetrics characteristics of the study women in Arba Minch HDSS site, February 15-March 15, 2019.

Respondent’s characteristics	Category	Frequency	Percentage
**Pre-pregnancy utilization of contraception (any modern methods) (n = 432)**	Yes	294	68.0
	No	138	32.0
**Knowledge about key pregnancy danger signs (n = 432)**	Not-knowledgeable	261	60.4
	Knowledgeable	171	39.6
**Birth order (n = 432)**	First	73	16.9
	Second	95	22.0
	Third	121	28.0
	Fourth and above	143	33.1
**Desire on recent pregnancy (n = 432)**:	Planned	333	77.1
	Not Planned	99	22.9
**Time for first ANC visit (n = 432)**	At or after 16 wks.	337	78.0
	Before 16 wks.	95	22.0
**Birth preparedness and complication readiness (BPCR) (n = 432)**	Not well prepared	301	69.7
	Well prepared	131	30.3

### Continuum of care for MNH services

#### Level of completion along continuum of care

**Fig 1:**
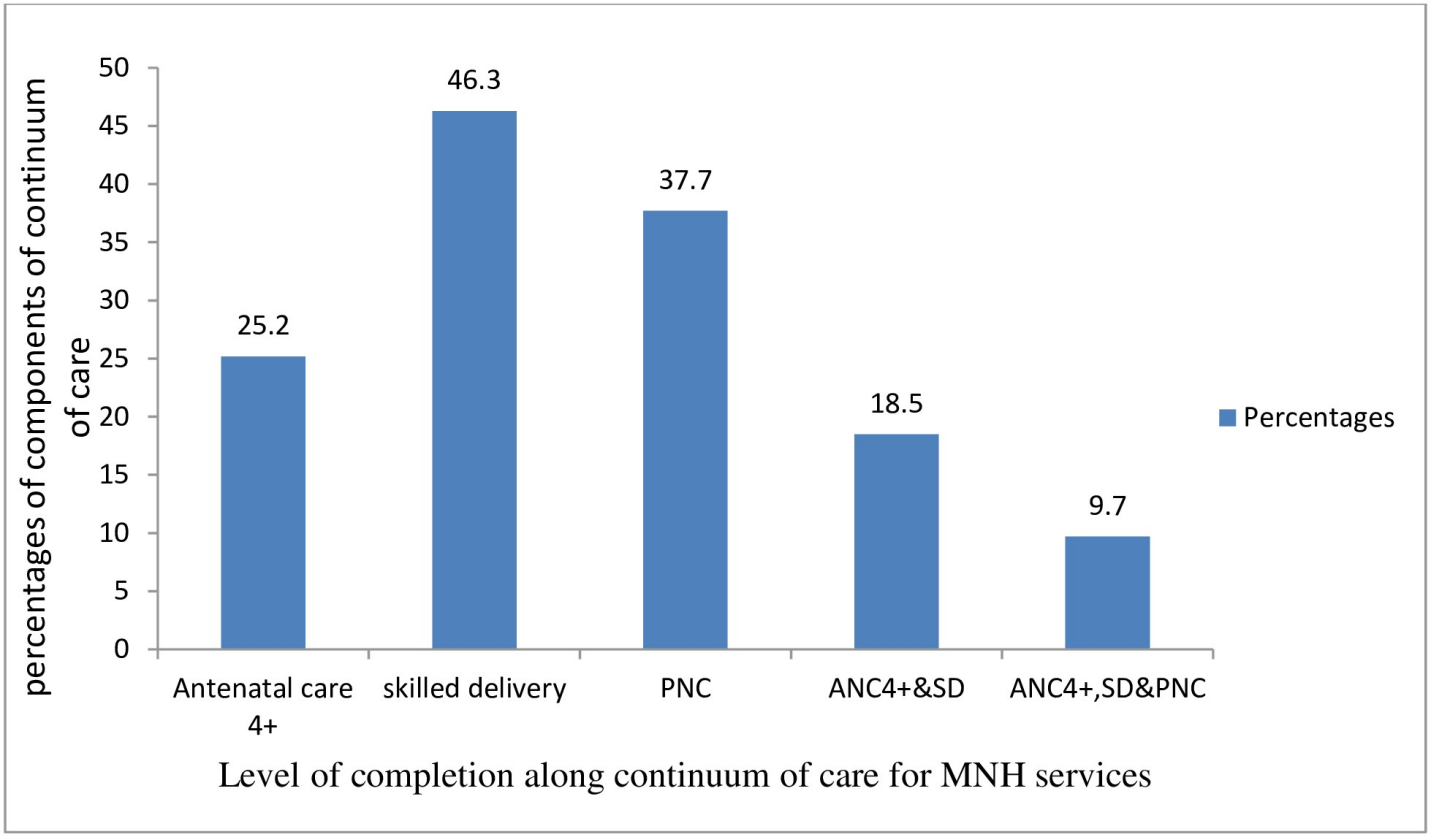
Level of completion along CoC for MNH services among women in Arba Minch DHSS site, February 15-March 15, 2019.

The overall prevalence of CoCamong the study women was 9.7% [95% CI: 6.9, 12.5] (Fig 1).

#### Antenatal care attendance during pregnancy

**Fig 2:**
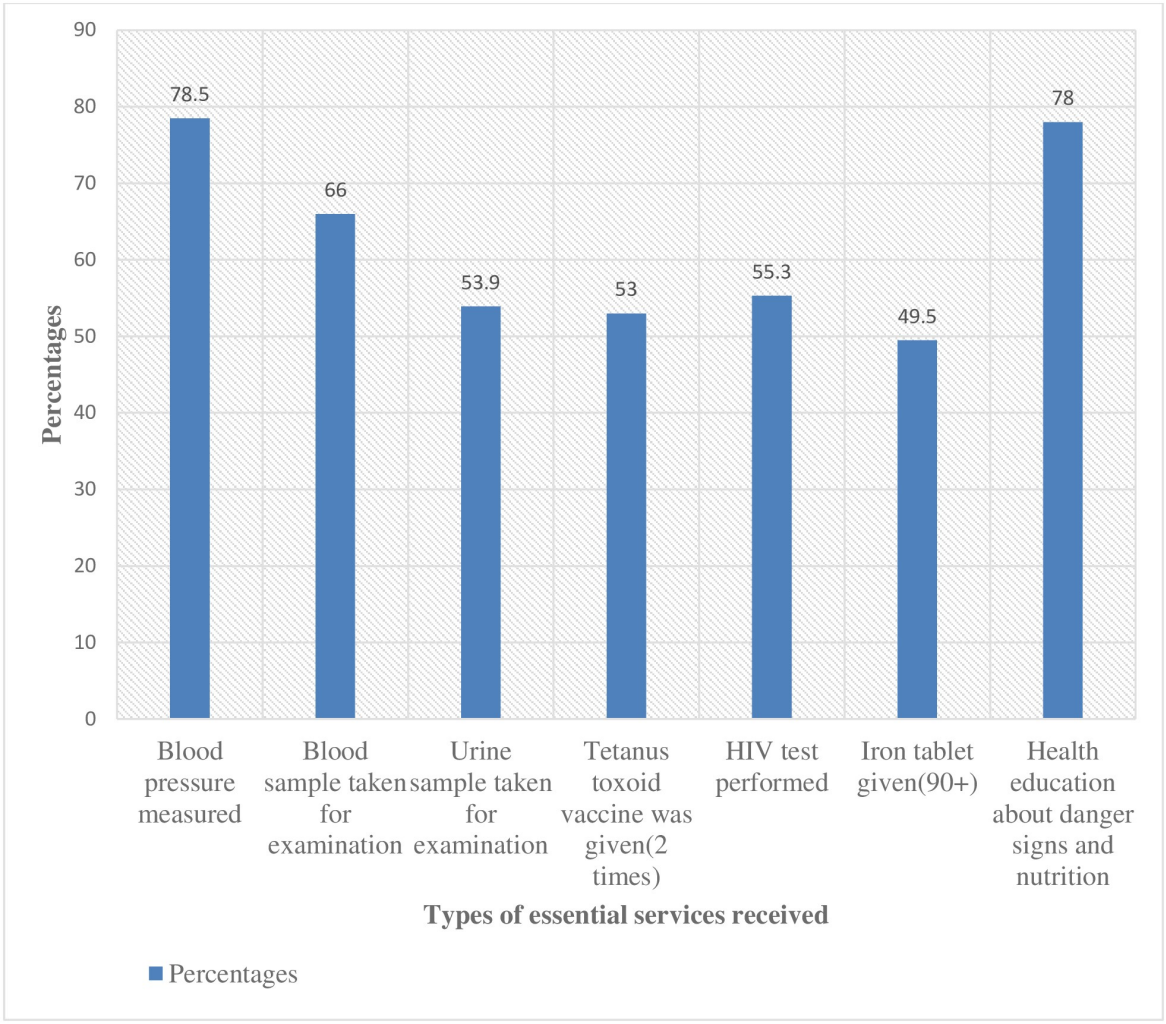
Percentages of the study women who received the essential ANC services in Arba Minch HDSS site, February 15-March 15, 2019.

**Fig 3:**
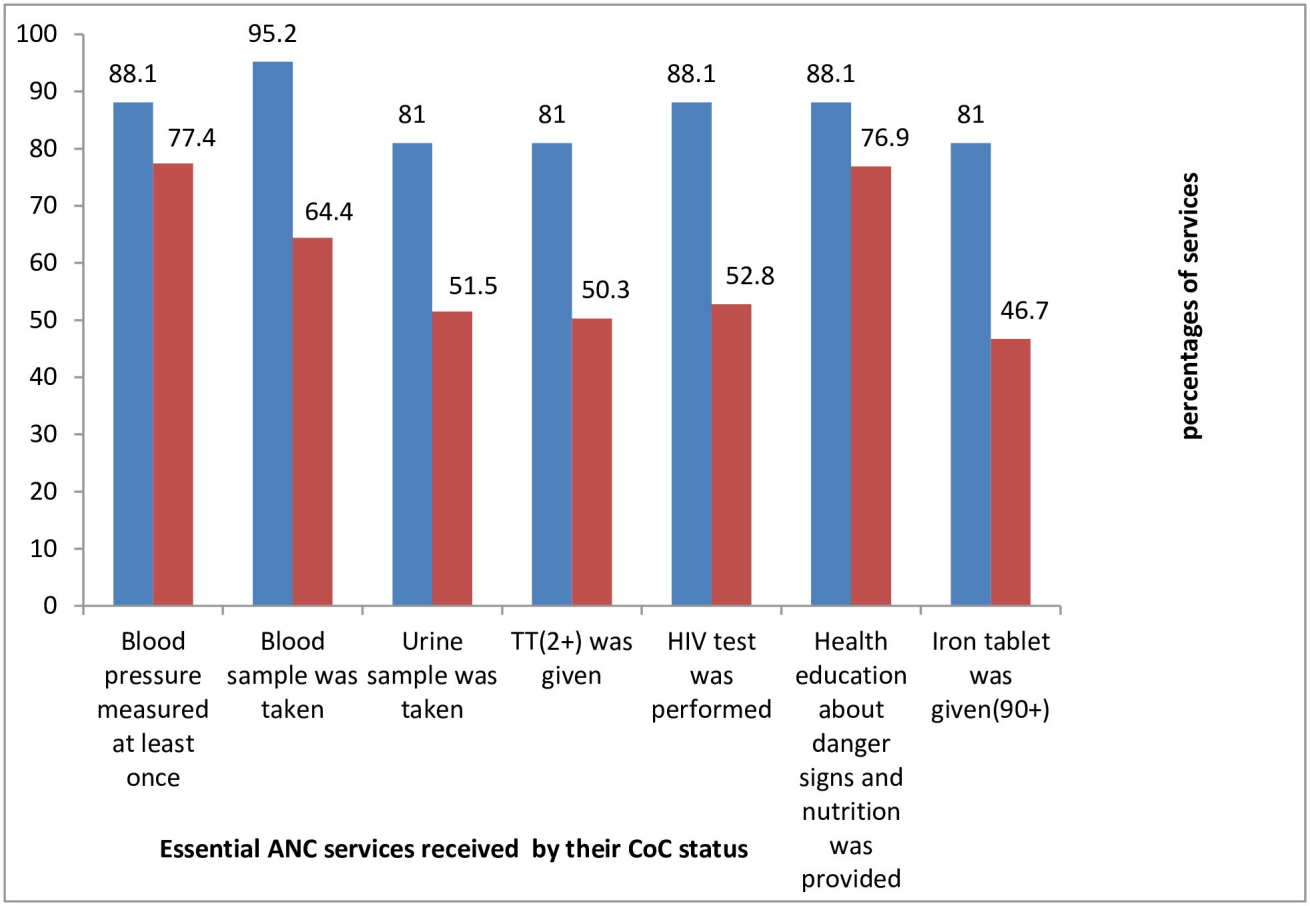
Percentages of the study women’s use of essential ANC services by their CoC status in Arba Minch HDSS site, February 15-March 15, 2019.

Among the women, 25.2% attended ANC for four or more visits (ANC4+) during their pregnancy time(Fig 1). Regarding the services provided during ante-partum period, among all respondents, 22% received the seven selected essential antenatal services (blood pressure measurement, blood and urine sample examination, vaccination for tetanus, HIV testing, provision of information on danger signs and nutrition and provision of iron tablets). The details of each service received and the content of care during the ANC areshown below (Fig 2) and (Fig 3) respectively.

#### Skilled birth attendance

Of 109 women with ANC4+ during ante-partum period, 73.4% gave birth by the assistance of a skilled birth attendant during the intra-partum period. This accounts only 18.5% of the total number of women in the study (Fig 1). Regarding MNH services provided during the intra-partum period, among women who delivered by the assistance of a skilled birth attendant, 93.8% had exercised skin-to-skin contact, and only 5% utilized immediate postpartum contraception during intra-partum period (Table 4).

**Table 4:** Content of intra-partum care provided to the study women in Arba Minch HDSS site, February 15-March 15, 2019.

Respondent’s characteristics	Frequency	Percentage
**Newborn services during inter-partum period (n = 80)**		
**Newborns had got weight measurement**	73	91.3
**Exercised skin-to-skin contact**	75	93.8
**Initiated breast feeding within one hours**	53	66.3
**Had got necessary immunization**	62	77.5
**Cord care**	74	92.5
**Maternal services during intra-partum period (n = 80)**		
**Blood pressure measured**	55	68.8
**Counseled about postpartum contraception utilization**	63	78.8
**Immediate post-partum contraception utilization**	4	5.0
**Counseled about signs of postpartum complication**	67	83.8

#### Postnatal care attendance

Regarding to postpartum period, out of 80 women with skilled birth assistance during the intra-partum period, 52.5% of them received post- natal care. This accounts to 9.7% of the total number of respondents (Fig 1). Among respondents who have had at least one visit for post-natal care after discharge from the health facility, 33.3% had their first PNC contact within two days, 45.2% attended PNC in 3–7 days, 14.3% attended PNC in seven days to two weeks, and 7.1% attended PNC in two weeks to six weeks postpartum(Table 5).

**Table 5:** Contents of PNC provided to the study women in Arba Minch HDSS site, February 15-March 15, 2019.

Respondent’s characteristics	Frequency	Percentage
**Newborn care services provided**		
**Cord care with chlorhexidine**	28	66.7
**Weight measurement**	20	47.6
**Counseling on infant feeding**	25	59.5
**Immunization**	27	64.3
**Maternal health care services provided**		
**Counseled about postpartum contraception**	39	92.9
**Utilized postpartum contraception**	18	42.9
**Counseled about signs of postpartum complication**	22	52.4
**Provided with iron tablet**	10	23.8

### Factors associated with completion along continuum of care

Multivariable analysis identified five independent factors affecting the completion status of MNH services. These were time for initiation of ANC, pre-pregnancy use of contraception, maternal employment, birth preparedness and complication readiness, and pregnancy desire.

The odds of completing CoC for MNH services was about 11 times higher for those who booked for ANC early (before 16 weeks) than their counterparts [AOR: 10.7, CI (5.1,22.7]. Similarly, the odds of completion of CoC was higher among women well prepared for birth and its complication during pregnancy than for those who were not well prepared [AOR: 2.9, CI (1.4, 6.1). Use of complete CoC for MNH services was significantly associated with, respondents who used contraception before their recent birth. The odds of CoC for MNH services were 4 times higher for those who used pre-pregnancy contraception when compared withtheir counterparts [AOR: 3.96, CI: 1.4,11.0] and employed women were had also higher odds of completion than non-employed women [AOR: 2.6, CI: (1.3,5.4)]. Women who planned for their recent pregnancy had also a higher odd of completion as compared to those who did not plan for their recent pregnancy [AOR:3.5, CI:(1.1,11.4)] (Table 6).

**Table 6:** Factors associated with CoC among the study women in Arba Minch HDSS site, February 15-March 15, 2019.

	CoC for MNH services			
Variables	Yes (%)	No (%)	COR(95% CI)	AOR(95% CI)
**Women’s education status(n = 432)**				
**Unable to read or write**	11(6.4)	162(93.6)	1	1
**Able to read or write**	6(11.8)	45(88.2)	1.9(0.7,5.6)^a^	1.9(0.5,6.9)
**Primary level of education**	17(11)	138(89)	1.8(0.8,4.0)^a^	1.8(0.6,5.4)
**Secondary and above**	8(15.1)	45(84.9)	2.6(1,6.9)^a^	1.9(0.5,7.6)
**Women’s employment status(n = 432)**				
**Non-employed**	20(7.5)	247(92.5)	1	1
**Employed**	22(13.3)	143(86.7)	1.9(1.1,3.6)^a^	2.6(1.3,5.4)^b^
**Perceived required time to reach health facilities**				
**> = 30 minutes**	11(6.1)	169(93.9)	1	1
**<30 minutes**	31(12.3)	221(87.7)	2.2(1.1,4.4)^a^	1.9(0.8,4.4)
**Pre-pregnancy utilization of contraception (n = 432)**				
**Yes**	37(12.6)	257(87.4)	3.8(1.5,9.9)^a^	3.9(1.4,10.9)^b^
**No**	5(3.6)	133(96.4)	1	1
**Knowledge on key pregnancy danger signs (n = 432)**				
**Not-knowledgeable**	18(6.9)	243(93.1)	1	1
**Knowledgeable**	24(14)	147(86)	2.2(1.2.4.2)^a^	1.6(0.7,3.7)
**Birth order**				
**First**	10(13.7)	83(86.3)	1	1
**Second**	10(10.5)	85(89.5)	0.7(0.3,1.8)^a^	0.7(0.3,2.3)
**Third**	10(8.3)	111(91.7)	0.6(0.2,1.4)^a^	0.5(0.2,1.5)
**Four and above**	12(8.4)	131(91.6	0.6(0.3,1.4)^a^	0.6(0.2,1.9)
**Desire on pregnancy (n = 432)**				
**Not planned**	4(4)	95(96)	1	1
**Planned**	38(11.4)	295(88.6)	3.1(1.1,8.8)^a^	3.5(1.1,11.4)^b^
**Time for first ANC booking (n = 432)**				
**At or after 16 wks**.	15(4.5)	322(95.5)	1	
**Before 16 wks**.	27(28.4)	68(71.6)	4.7(2.5,9.2)^a^	10.7(5.1,22.7)^b^
**BPCR (n = 432)**				
**Not-well prepared**	20(6.6)	281(93.4)	1	1
**Well Prepared**	22(16.8)	109(83.2)	2.8(1.5,5.4) ^a^	2.9(1.4,6.1) ^b^

COR = Crude Odds Ratio, AOR = Adjusted odds ratio

^a^ significant in bivariate analysis at p-value of less than 0.25,

^b^ statistically significant association at p-value of less than 0.05 after adjusting for all the other variables in the model- BP/CR, time for ANC initiation, desire on pregnancy, perceived required time to reach health facilities, women’s employment status, women’s education status, pre-pregnancy family planning utilization, knowledge on key pregnancy danger signs, and birth order.

## Discussion

The level of CoC for MNH services was very low in the study area. Socio-demographic and maternal health care service-related factors of the study women were independently associated with the completion of the CoC. Women’s employement, early initiation of ANC, receipt of pre-pregnancy maternity care, being well-prepared for birth and its complications and planning for pregnancy remained the independent factors associated with the completion of CoC.

The magnitude of CoCis in agreement with a previous study in Tanzania [17]; however, it is higher than a study conducted in Cambodia [38], and lower than studies conducted in Pakistan and Egypt [32,37]. Even though a disproportion in the level of completion exists among countries, the general figure implies a significantly low devotion of women in completing the CoC for MNH services. The low level in Ethiopia could be explained by the low level of timely initiation and completion of ANC [43–45], the high magnitude of home birth [27, 46, 47]and the low uptake of PNC [27, 29]. This implies that large number of women dropped from utilization of the MNH services and at risk of preventable maternal and newborn deaths that would have been averted had women had got the interventions proven to reduce those deaths.

Women’s employment status was associated with CoC for MNH services. Women who were employed were having a higher odd of completion in the CoCas compared with those who were not employed. This finding is in line with prior studies conducted in Ethiopia [22] and Egypt [37]. The possible reason might be due to the fact that employed women would have a control to their economy and therefore be more likely autonomous to decide on their own health care as compared with those who were unemployed [21, 30, 48, 49]. However, a study from Pakistan had a contrasting finding [32] where secondary data analysis found that employed women had a lower odds of completion in the CoC as compared with unemployed women. The discrepancy in the two studies might be due to the fact that the proportion of women who make autonomous decision in seeking for healthcare in this study was quite higher than those women in the Pakistan study [21]. Thus, women empowerment through employments could improve uptake and determination to MNH services [50–53].

Time for ANC initiation was one of the factors that facilitate CoCin this study. This is plausible because women who present early for ANC have greater chance to completing the recommended optimum ANC visits and thus complete the first continuum required for completion of CoC. WHO also recommends on early initiation of ANC in order to attain adequate ANC visits and improve women’s health [54]. The reason for this finding might be early initiation of ANC gives the opportunity for health promotion, a good opportunity to be informed about pregnancy danger signs and obstetric complications and isa powerful predictor of the content and quality of ANC services, [45, 55, 56]. In this study, among women who initiated ANC early, around two-third received full essential ANC services and more than two thirds attended antenatal care four or more times. We suggest that early commencement of ANC is vital to completion of the ANC continuum as well as along the three continuums of MNH services. This has an inference to provision of quality ANC in the area and by extension in the country.

Another finding is the association between contraceptive utilization before recent birth and completion in the CoC for MNH services. This finding was supported by study conducted in Bangladesh where the odds of having skilled birth attendancewere higher for women who used pre-pregnancy contraception when compared with women who did not use pre-pregnancy contraceptive [11]. The reason behind this might be that the currently existing service integration between pre-conception care and the CoC for MNH services would give women the chanceto have informationabout subsequent maternal and newborn services and might have helped them to enter early to prenatal care as they can set plans with health professionals on the service continuum [57]. The strengthening of preconception care like counseling and provision of family planning and other reproductive health services, which the WHO has set as a strategy to reduce maternal and child morbidity and mortality, closes the gap between reproductive components and maternal health components of CoC [57]. Thus, the concerned health authorities should strengthen the implementation of preconception care in order to achieve the targets of national health sector transformation plan and to achieve the sustainable development goal 3 targets [10]. In order to achieve the CoC and get the maximum benefits from MNH services, this finding directs to enhanced efforts in the integration of maternal and child health services.

Birth preparedness and complication readiness is one of factors positively associated with completion in the CoC. Women who were well-prepared for birth and its complications had higher odds of completion as compared with their counterparts. This finding is a new addition of this study and has a policy implication as BPCR is one of twelve WHO recommendations for health promotion in order to increase the use of skilled care at birth and to increase the timely use of facility care for obstetric and newborn complicationsduring postnatal period [58]. Women who were well prepared for birth and its complications were likely to be educated, with higher socio-economic class and having a better knowledge on maternal and newborn danger signs and thus in favor to complete the CoC for MNH services [39, 40, 59, 60]. Despite its greater implication in this study, more than half of women who completed the CoC for MNH services were less prepared for birth and its complications. A previous study in the same setting also showed a lower practice of BPCR among pregnant women [60]. These outcomes call for a further attention on improvements of women’s knowledge and practice on BPCR.

The odds of completion in the CoC among women who planned the recent pregnancy was higher when compared to their counterparts in the present study. The finding is inconsistent with findings from Ghana in 2015 [18] though a related study from Ghana in 2018 found no association between pregnancy intention and CoC for MNH services [19]. The inconsistence with the former study by Yeji F et al [18] might be due to the difference in proportion of unplanned pregnancy between the two studies where the Ghanan study has higher proportion of women with unplanned pregnancy. The possible reason for the association of CoC with planning for pregnancy in this study would be owing to the higher proportion of women who initiated ANC early among women with planned pregnancy than those with unplanned pregnancy (17.8% versus 4.2%). This can be explained by the fact that prior planning on pregnancy would reduce delay in prenatal care which might increase the chance of frequent visits along the CoC [43, 61]. Therefore, a concerted effort is needed to reduce unplanned pregnancy and improve CoC along MNH services so that women and new born can get the maximum benefit from the range of services.

Likewise, almost all the unplanned pregnancies wereamong unmarried women in our study. Such women may initially attempt to deny their pregnancies to themselves and to conceal them from others since most of the time premarital pregnancies were highly stigmatized [62]. As a result, women may become less motivated to seek care along theCoC compared withthosewith planned pregnancy. Even though the present study was informative in looking to individual and household level factors deemed to affect the level of CoC, it was not free from limitations. First, the study included only women who gave birth in the previous year in the HDSS site by excluding women who did not receive ANC at all. Since such women wouldpossibly be familiar with pregnancy care through the surveillance system, there would be overestimation of women who completed the CoC in the study area. Second, the study was based on self-reports and this might have introduced social desirability bias. Moreover, the sampling frame was based on secondary data which may not have registered all eligible women for the study. Owing to resource restrictions, the study was limited in a small locality and this might make it to fall short of generalizability to a wider population. Longitudinal studies covering larger populations and focusing on other contextual factors beyond the individual women and their families would give better findings for the improvement of women’s completion status and subsequently the improvements in maternal and child health.

## Conclusion

In this study, the coverage of CoC was lower when compared with other studies. The main identified factors associated with completion of the CoC for MNH services were pre-pregnancy contraceptive use, pregnancy desire, birth preparedness and complication readiness, women’s employment status and timing for antenatal care booking. Despite their higher contribution to CoC for MNH services, the proportion of early initiation of ANC and provision of essential selected elements of antenatal care services were very low. Thus, strengthening efforts in improving early initiation of antenatal care, reducing unplanned pregnancy and promotion of birth preparedness and complication readiness interventions is critical in achieving the CoC for MNH services. This calls for further promotion of educational programs, behavioral change communication activities targeting mothers and families, and advocacy at higher levels.

## Supporting information

S1 Fig Schematic presentation of the sampling procedure. (TIF)

S1 Data The raw data supporting the findings of this article. (XLSX)

S1 Questionnaire English version questionnaire. (DOCX)

S1 File Additional definitions and measurements. (DOCX)

S2 File Bivariate binary logistic regression. (DOCX)
