# Supplementary figures and images for: Level of completion along continuum of care for maternal and newborn health services and factors associated with it among women in Arba Minch Zuria woreda, Gamo zone, Southern Ethiopia: A community based cross-sectional study
Source: PLoS One. 2020 Jun 8;15(6):e0221670. doi: 10.1371/journal.pone.0221670 (PMC7279583; doi:10.1371/journal.pone.0221670)

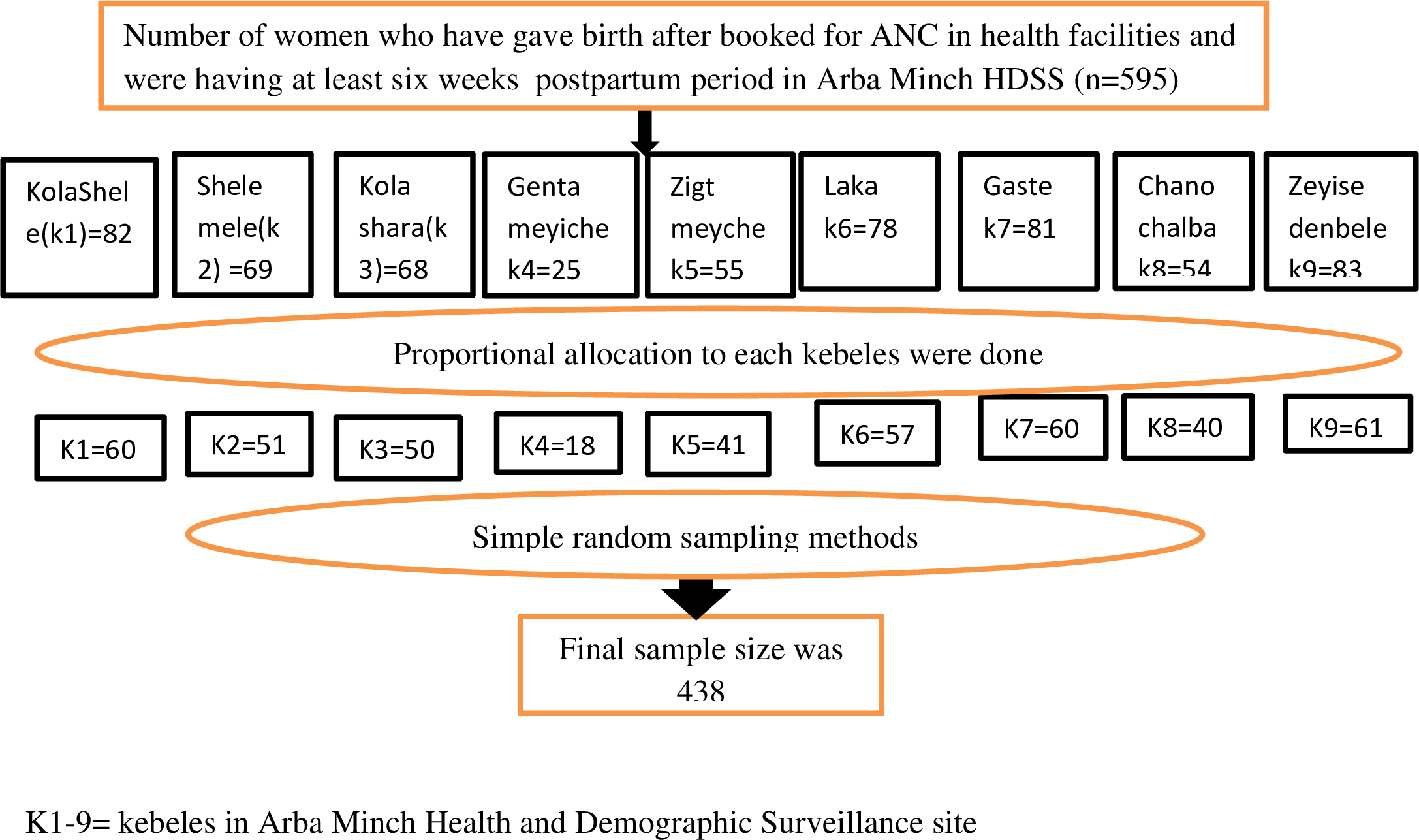

Supplement: S1 Fig — (TIF) [file pone.0221670.s001.tif]
