# Supplementary material for: Level of completion along continuum of care for maternal and newborn health services and factors associated with it among women in Arba Minch Zuria woreda, Gamo zone, Southern Ethiopia: A community based cross-sectional study
Source: PLoS One. 2020 Jun 8;15(6):e0221670. doi: 10.1371/journal.pone.0221670 (PMC7279583; doi:10.1371/journal.pone.0221670)
Supplement: S1 Questionnaire — (DOCX) [file pone.0221670.s003.docx]

## English version Questionnaire

## Information Sheet

Good Morning/Afternoon, My name is ________________________. I am a trained data collector for the research, level of completion along continuum of care for MNH and factors associated with it among women who have recently gave birth after booked for ANC in the health facilities in Arba Minch Health and Demographic Surveillance Site, Gamo Zone, Southern Ethiopia, 2018/2019. This study will be conducted by Dereje Haile who is a Master of public health student in Arba Minch University. You are selected as a matter of chance; and nothing special is attached to it. The interview will take about 30 minutes. If you participate in the study, there will not be any harm to you or your family. Your name and other personal identifiers will not be recorded on data collection form and the information that you give us will be kept confidential. The information you provide will not be used for another purpose than this study. For this interview, there will not be an immediate benefit in terms of money; rather the information you provide will help the government and concerned bodies to improve level of completion along continuum of care for MNCH services which can also benefit you and your community. Your participation is voluntary and you have the right to stop the interview at any time. Your decision about not to participate is respected. If you have any question on what we have talked so far you welcome. If you have any question at any time you can contact the principal investigator through the following address:

Dereje haile Mobile No: +251910966192; Email: derehaile2010@gmail.com

## Annex 2. Consent Form

I hope, you understand the objectives of this study. So that, are you willing to participate in this study? 1. Yes 2. No. If yes, thank you for your willingness.

## Annex 3. English version questionnaire

English version semi-structured questionnaire, study questionnaire prepared on level of completion along continuum of care for MNH and associated factors among women who have recently gave birth after booked for ANC in health facilities in Arba Minch Health and Demographic Surveillance Site, Gamo Zone, southern Ethiopia, 2018/2019.

English Version Questionnaire for women’s

| S,no | Questionnaire Code______  **Instruction: Circle the appropriate answer** provided and where applicable writes the required responses in the spaces provided.  PART ONE   - 1. Socio-Demographic questions | Response | Skip |
| --- | --- | --- | --- |
| 101 | How old are you? | Completed age------1  I don’t know--------99 |  |
| 102 | What is your educational status? | Unable to read and write---1  Able to read or write------2  Primary education(1-8)----3  secondary ---4  colleges--------5 |  |
| 103 | What is your occupation? | Housewife-------1. Farmer-------2  Merchant-----3. Student-------4  Government Employee----5. Daily laborer----6  Other (specify)----7 |  |
| 104 | What is your marital status? | Single--1, Married-----2. Divorced---3, Separated---4. Widowed-------5, |  |
| 105 | \| What is you religion? \| Orthodox ------1, protestant------2  Catholic -------3, Muslim -----4  Others --------999 \| \| --- \| --- \| | Orthodox ---1, Protestant----2  Muslim-----3, Others--------4 |  |
| 106 | What is your husband’s educational status? | Unable to read and write---1  Able to read or write------2  Primary education(1-8)------3  secondary ----4  college--------5  I don’t know-------99 |  |
| 107 | What is your husband’s occupation? | Farmer-------1  Merchant-----2  Student-------3  Government Employee----4  Daily laborer----5  Other (specify)----6 |  |
| 108 | Who were decision maker for the health care seeking? | Husband only---1, a woman alone----2 Both-------3, family ----4 |  |
| 109 | Is there any nearby Health institution in your kebele? | Yes----1, No------2, I don’t know------99 |  |
| 110 | What did you think about distance to health facilities? | Long---1, medium-----2, short------3. I don’t know----99 |  |
| 111 | How long does it take to go the nearest health institution? | <30 min---1, >30 min----2. I don’t know------99 |  |
| 112 | What was your mode of transportation to health facilities? | On foot----1, By motorcycle/bicycle/------2.  By car -----3, Other ------4 |  |
| 113 | Had you heard about MNCH service? | Yes---1, No------2 | If yes ans, next Qno.  114-115 |
| 114 | From where? | By radio/TV--1, from health professional---2. School --3. Friends----4. Relatives ------5, Others---------6 |  |
| 115 | If you heard by radio/TV, how often? | Always ----1. Once in a week---2, more than once a week---3, |  |
| 116 | Had you heard about CBHI? | Yes ---1, No----2 | If yes ans. Qn. 117 |
| 117 | Had you ensured in CBHI? | Yes----1, No-----------2 |  |

| - 1. **Socioeconomic: Wealth index measurement** | |  | | | | |
| --- | --- | --- | --- | --- | --- | --- |
| 118 | Does your household have: |  | Response category | | | Code |
|  |  | | Yes | No | |  |
|  | 118.1. Electricity | | 1 | 2 | |  |
|  | 118.2. Radio | | 1 | 2 | |  |
|  | 118.3. Television | | 1 | 2 | |  |
|  | 118.4. Non-mobile telephone | | 1 | 2 | |  |
|  | 118.5. A table | | 1 | 2 | |  |
|  | 118.6. A chair | | 1 | 2 | |  |
|  | 118.7. Bed | | 1 | 2 | |  |
| 119 | Does anyone of your household  member own: | | Yes | No | |  |
|  | 119.1. Does anyone of your household member own: Watch | | 1 | 2 | |  |
|  | 119.2. Does anyone of your household member own: Mobile phone | | 1 | 2 | |  |
|  | 119.3. Does anyone of your household member own: Bicycle | | 1 | 2 | |  |
|  | 119.4. Does anyone of your household member own: Motorcycle? | | 1 | 2 | |  |
|  | 119.5. Does anyone of your household member own: Bajaj | | 1 | 2 | |  |
|  | 119.6. Does anyone of your household member own: An animal-drawn cart? | |  |  | |  |
|  | 119.7. Does anyone of your household member own: A car/truck? | |  |  | |  |
| 120 | Main material of the floor(observe) | | Yes | | No |  |
|  | 120.1 Cemented | |  | |  |  |
|  | 120.2. Others(Mud/crow dung, Earth/Sand, Wood, Ceramic) | |  | |  |  |
| 121 | What is the main source of drinking water for your household? | | Yes | | | No |
|  | 121.1. Piped water | |  | | |  |
|  | 121.2. Others (Dug well, Water from spring, Rainwater, Tube well or borehole, Lake /pond/stream/canal) | |  | | |  |
| 122 | What type of fuel does your household mainly use for Cooking? | | Yes | | | No |
|  | 122.1. Electricity | |  | | |  |
|  | 122.2. Natural gas | |  | | |  |
|  | 122.3. Biogas | |  | | |  |
|  | 122.4. Kerosene | |  | | |  |
|  | 122.5. Charcoal | |  | | |  |
|  | 122.6. Wood | |  | | |  |
|  | 122.7. Straw /shrubs/grass | |  | | |  |
|  | 122.8. Agricultural crop | |  | | |  |
|  | 122.9. Animal dung | |  | | |  |
|  |  | | Yes | | | No |
| 123 | Does any member of this household have a bank or microfinance saving account | |  | | |  |
| 124 | Does your household own? | | Yes | | | No |
|  | 124.1. Does your household own milk Cows? | |  | | |  |
|  | 124.2. Does your household own oxen? | |  | | |  |
|  | 124.3. Does your household own hen? | |  | | |  |
|  | 124.4. Does your household own Goat or Sheep? | |  | | |  |
|  | 124.5. Donkey or Horse? | |  | | |  |
|  | 124.6. Does your household own Mule? | |  | | |  |
| 125 |  | | Yes | | | No |
|  | Does this household own any agricultural land? | |  | | |  |
| 126 | How many hectares of agricultural land? | | Yes | | | No |
|  | Less than 1 hectar  Greater than one hectar | |  | | |  |
|  |  | | Yes | | | No |
| 127 | Did you rent/lease out land over the last 12 months? | |  | | |  |
| 128 | In the past 12 months, how many Kilo grams (quintals) of crops did you produce/harvest in total? | | Yes | | | No |
|  | Less than 5 | |  | | |  |
|  | More than five | |  | | |  |

| **S.no** | **Part two: Obstetric history related questions** | **Response** | | | | | **Skip** |
| --- | --- | --- | --- | --- | --- | --- | --- |
| 201 | Had you heard about family planning? | Yes ---1, No-----2 | | | | | If yes ans. Qn202 |
| 202 | Had you history of pre-pregnancy utilization of family planning (any method)? | Yes----1, No-------2 | | | | |  |
| 203 | How old were you when you got pregnant for the first time? | Completed age------------1.  I don’t know---99 | | | | |  |
| 204 | How many pregnancies did you have? | ---------------------------------- | | | | |  |
| 205 | What was your desire on pregnancy? | Planed ---1, Un planed---2, mistimed--3 | | | | |  |
| 206 | What was the frequency of ANC visit during pregnancy? | One ANC-----1, two ANC ----2, three ANC visit------3, >=4 ANC--------4, I don’t know---99 | | | | |  |
| 207 | How old was your pregnancy when you started antenatal visits? | ------------------------  I don’t know-----99 | | | | |  |
| 208 | Where did you attend your antenatal visits? | 1. Hospital 2. Health center  3. Health post 4. Own home | | | | |  |
| 209 | What type of professional personnel provides the antenatal care service? | 1. Doctor/Health Officers/Nurse  2. Health Extension Worker  3. Health professional but I don’t know his/her title.  99. I don’t know | | | | |  |
| 210 | Did you remember about the services provided during ANC? | Yes-----------1. No ---------------2 | | | | | If no skip to 213 |
| 211 | If yes, was the recommended ANC service provided during follow-up?  (More than one ans. Possible) |  | | | | |  |
|  |  | Yes | No | | | |  |
|  | Blood pressure measured? |  |  | | | |  |
|  | Blood sample was taken? |  |  | | | |  |
|  | Urine sample was taken? |  |  | | | |  |
|  | TT2+ vaccination was provided? |  |  | | | |  |
|  | HIV test was provided? |  |  | | | |  |
|  | Health education was provided on (danger signs, nutrition, and birth preparedness)? |  |  | | | |  |
|  | Iron provision 90+ tablet? |  |  | | | |  |
| 212 | Did you know the presence of birth preparedness and complication readiness? If yes what are they?  (More than one ans. Possible) | **Yes** | **No** | | | |  |
|  |  |  |  | | | |  |
|  | Identified place of birth? |  |  | | | |  |
|  | Identified birth attendants? |  |  | | | |  |
|  | Identified the location of closest health facilities for birth? |  |  | | | |  |
|  | Prepared emergency transportation? |  |  | | | |  |
|  | Saved money for emergency? |  |  | | | |  |
|  | Identified labor and birth companion? |  |  | | | |  |
|  | Identified potential blood donor? |  |  | | | |  |
|  | Identified support person to look after the home and other children while women away? |  |  | | | |  |
| 213 | Did you have any information on danger signs of pregnancy? | Yes----1,. No-----------2. | | | | | if yes ans. 215 |
| 214 | If your answer is yes, what are they?  (multiple response are possible)  Vaginal bleeding--------------1.  Severe headaches—---------2  Blurring of vision--------------------3.  Severe lower abdominal pain----4. | Fast breathing---5.  High grade Fever---6.  Swelling of face or legs-----7.  Mention others --------- | | | | |  |
| 215 | What was the place of delivery? | Health facility----1, Home------------2 if at home Ans. Q no. 225 | | | | |  |
| 216 | What services provided during childbirth?  ( more than one answer possible)? | **Newborn services** | | **Yes** | | **No** |  |
|  |  | Exercised skin to skin contact | |  | |  |  |
|  |  | Cord care | |  | |  |  |
|  |  | Initiated breast feeding with in one hours | |  | |  |  |
|  |  | Immunization | |  | |  |  |
|  |  | Weight measurement | |  | |  |  |
|  |  | Others ------------ | |  | |  |  |
|  |  | No service at all | |  | |  |  |
|  |  | **Maternal services** | | **Yes** | | **No** |  |
|  |  | Blood pressure measurement | |  | |  |  |
|  |  | Counseling on postpartum complication | |  | |  |  |
|  |  | Counseling on Postpartum FP | |  | |  |  |
|  |  | Provision of postpartum family planning | |  | |  |  |
|  |  | Others ------------------- | |  | |  |  |
|  |  | No service at all | |  | |  |  |
| 217 | Did you receive professional assistance while you gave your last birth? | Yes ------1, No----------2 | | | | |  |
| 218 | What was the mode of delivery of this child? | Spontaneous vaginal delivery-----1  Instrumental/Cesarean delivery-----2 | | | | |  |
| 219 | Who attended the delivery? | Doctor/nurse/midwife/health officer-1  Community HEW------------------2  Non health professional--------------3  Traditional birth attendants---------4  I don’t know----------------99 | | | | |  |
| 220 | What was the birth order of the last child? | ---------------------- | | | | |  |
| 221 | Did you have any information on danger signs of pregnancy? | Yes------1, No-----------------2 | | | | |  |
| 222 | If your answer is yes, what are they?  (multiple response are possible)  Severe Vaginal bleeding--------------1.  High grade fever—---------2  Foul smelling vaginal discharge------3.  Urinary incontinence----4.  Breast pain-----------------5 |  | | | | |  |
| 223 | Had you attend postpartum health care service? | 1. Yes 2. No | | | | | If no skip to Q.234 |
| 224 | If yes for what purpose you were attended PNC? | 1. simply for checkup 2. because I was sick 3. because my child was sick at the time 4. others | | | | |  |
| 225 | When did you attend first PNC visit after child birth? | Within two days--1,  three days to seven days----2  seven days to two weeks—3,  two weeks to six weeks---4, ,  I don’t know-99 | | | | |  |
| 226 | Where did you attend your postpartum health care service? | 1. Hospital 2.Health center 3. Health post 4. Own home | | | | |  |
| 227 | How many times you attended PNC? | One times---1. Two times---2.  Three times--3. More than three times----4.  I don’t know—99 | | | | |  |
| 228 | What type of professional personnel provides the postnatal care service? | 1. Doctor 2. Health Officers  3. Nurse 4. Health Extension Worker  5. Health professional but I don’t know her/his title.  99. I don’t know | | | | |  |
| 229 | What services was given to you and your newborn? (more than one answer possible) | **Newborn service** | | | **yes** | **No** |  |
|  |  | Cord care | | |  |  |  |
|  |  | Received necessary immunization | | |  |  |  |
|  |  | Weight measurement | | |  |  |  |
|  |  | Counseling on child feeding | | |  |  |  |
|  |  | Mention other | | |  |  |  |
|  |  | No service at all | | |  |  |  |
|  |  | **Maternal service** | | | **yes** | **No** |  |
|  |  | Counseling on family planning | | |  |  |  |
|  |  | Provision of family planning | | |  |  |  |
|  |  | Counseling on postpartum complication | | |  |  |  |
|  |  | Received anemia treatment | | |  |  |  |
|  |  | Mention others---------------- | | |  |  |  |

| **Amharic version questionnaire**  **የመረጃ ቅጽ**  በባለፉት 12 ወራት ውስጥ በአርባ ምንጪ ዙርያ ወረዳ በተመረጡ ቀበሊያት በወለዱ እናቶች ላይ የነበረው የተማላ የእናቶችንና የጨቅላ ህፃናት አገልግሎት አጠቃቀም ያለበት ደረጃና ተያያዥ ሁነቶች ፣ ጋሞ ዞን/ደቡብ ኢትዮጲያ፣2011 ዓ.ም  ሰላምታ፡-ጤና ይስጥልኝ!! እኔ------------እባላለሁ፡፡ ይህ ጥናት የሚካሄደው አቶ ደረጀ ኃይሌ በተባሉት ሲሆን በዩኒቨርሲቲው ሙሉ ፈቃድ ታግዘው የሁለተኛ ዲግሪያቸው መመረቂያ ጽሁፍ በወረዳው በተመረጡ ቀበሌዎች ላይ ያለው የተማላ የእናቶችንና የጨቅላ ህፃናት አገልግሎት አጠቃቀም ያለበት ደረጃ ለመለየትና ከአገልግሎቱ ጋር በተያያዘ ያሉ ተያያዥ ሁነቶችን ለመለየት ነው፡፡ይህን መጠይቅ ለመሙላት ከ25 እስከ 30 ደቂቃ የሚወስድ ሲሆን በዚህ ቃለ መጠይቅ ፈቃደኛ ካልሆኑ ያለመሳተፍ መብት አለዎት፡፡ በዚህም ምክንያት በእርስዎም ላይ ሆነ በቤተሰቦት ላይ የሚደርሰስ ምንም ዓይነት ችግር የለም፡፡በተጨማሪም የሚሰጡት መረጃ ከተባለለት ጉዳይ ውጪ እንደማይውል እና ሚስጥራዊነቱ የተጠበቀ እንደሚሆን አረጋግጣለሁ፡፡በቃለ መጠይቅ ወቅት ለእርስዎ ግልጽ ያልሆነ ነገር ካለ መጠየቅ ይችላሉ፡፡ ለመመለስ ፈቃደኛ ያልሆኑበት ጥያቄ ካለም ማለፍ ይችላሉ፡፡ በየትኛዉም ምክንያት በመጠይቁ መሃል ማቋረጥ ቢፈልጉ ጥያቄዉን የማቋረጥ መብት አለዎት፡፡  ተጨማሪ ጥያቄ ካለዎትና እንዲሁም በቀጣይ የተሰበሰበው መረጃ ውጤት ለማወቅ ከፈለጉ ከዚህ በታች በተጠቀሰው የዋናው የጥናቱ ባለቤት ስልክ ቁጥርና አድራሻ ተጠቅመው ማግኘት ይችላሉ፡፡  እርሶም በዚህ ጥናት በመሳተፎ ከልብ አመሰግናለሁ!!!!!!  ደረጀ ኃይሌ  ስልክ ቁጥር +251910966192  አድራሻ derehaile2010@gmail.com  **የስምምነት ቅጽ**  ከሊይ የተፃፈውን የመረጃ ቅፅ አንብቤ የጥናቱን አላማና ጥቅም በግልጽ ተረዴቻለሁ፡፡በዚህም መሰረት  ያለጥናት ቡድኑ አባላት ተፅእኖ በሙለ ፈቃደኝነት በዚሁ ጥናት በመሳተፍ በአርባ ምንጪ የጠናና የህብረተሰብ ጉዳዮችን የምዳሰስ መረጃ ምሰበሰቢቤት ቀበሊያት ያለው የተማላ የእናቶችና የጨቅላ ህጻናት አገልግሎት ተጠቃሚነትና ተያያዥ ጉዳዮች ዙሪያ ያለው ሁኔታ ለመለየት በሚደረገው ጥናት ላይ የሚጠበቅብኝን አስተዋፅኦ ለማበርከት መወሰኔን በፊርማዬ አረጋግጣለሁ፡፡  የተሳታፊው ቁጥር ________**_______** ፊርማ **______________**ቀን___________  የመረጃ ሰብሳቢ ሥም ________________________ ፊርማ ________________  መረጃ የተሰበሰበበት ቀን____________የተጀመረበት ሠዓት ______ያለቀበት ሰዓት _______  የተቆጣጣሪ ሥም ______________________ ፊርማ_____ ቀን_____________ | | | |  |
| --- | --- | --- | --- | --- |
| S,no | የ ጥ.ቁ መለያ-------------------  ምድብ፤1 የማህበረሰብና የኢኮኖሚ ጉዳዮችን የሚዳስስ መጠይቆች፡፡ | ምላሽ |  | |
| 101 | ዕድመሽ ስንት ይሆናል? | ሙሉ እድመ-------------- አላቅም----------9 |  | |
| 102 | የትምህርት ደረጃሽ ስንት ይሆናል? | ማንበብና መጻፍ የማይችል-----1  1-8ኛ ክፍል የተማረ----2  9ኛ ና ከዛ በላይ ---3 |  | |
| 103 | የስራ ድርሻሽ ምንድነወ? | የቤት ዕመበት-------1 .አርሶ አደር----2  ነጋዴ----3. ተማር-------4.  የመንግስት ሠራተኛ----5  የቀን ሠራተኛ----6. ለላ ካለ ይጠቀስ---- |  | |
| 104 | የትዳር ሁነታ ልነግሩን ይችላሉ? | ያላገባ--1, ያገባ-----2  የተፋታ---3, የሞተባት-------4 |  | |
| 105 | \| ሃይማኖትሽ ምንድነዉ? \| Orthodox ------1, protestant------2  Catholic -------3, Muslim -----4  t  r  ---- -- 9 9 \| \| --- \| --- \| | ኦርቶዶክስ---1, ፐንጤ--2  እስልምና-----3, ለሎች-------- |  | |
| 106 | የባለበትሽ የትምርት ደረጃ ልነግሩን ይችላሉ? | ማንበብና መጻፍ የማይችል-----1  1-8ኛ ክፍል የተማረ----2  9ኛ ና ከዛ በላይ ---3  ኣላቅም--------9 |  | |
| 107 | የባለበትሽን የስራ ድርሻ ልነግሩን ይችላሉ? | አርሶ አደር----1 ነጋዴ----2. ተማር----3  የመንግስት ሠራተኛ--4. የቀን ሠራተኛ----5  ለላ ካለ ይጠቀስ---- |  | |
| 108 | የህክምና አገልግሎት ለማግኘት እቤት ዉስጥ ዉሳነ ሰጭ አካል ማን ነዉ? | ባል ቢቻዉን-1, ምስት ብቻዋን---2  ሁለቱም-------3,የበተሰብ አባል ----4 |  | |
| 109 | የጤና አገልግሎት ለማግኘት ጤና ተቋማት በ አቅራቢያ ይገኛል? | አዎን---1, የለም-----2, አላቅም-----9 |  | |
| 110 | አዎን ካሉ፡ ስለ ርቀቱ ምን ያስባሉ? | ሩቅ---1 መካከለኛ---2, ቅርብ----3. አላቅም-9 |  | |
| 111 | ወዴ ጤና ተቋም ለመድረስ በግምት ምንያህል ሳት ይጨርሳል? | ከ30 ደቅቃ በታች---1, ከ30 ደቅቃ በላይ-2  አላቅም--9 |  | |
| 112 | ወደ ጤና ተቋም ስሔዱ በምን ይሔዳሉ? | በእግር----1. በሞተር----2.  በመክና -----3. ሌላ ይጠቀስ ------ |  | |
| 113 | ስለ እናቶችና ህጻናት የጤና አገልግሎት ሰምቶ ያዉቃሉ? | አዎን----1, አላዉቅም-----------2 | መልሶ አዎን ከሆነ ጥ.ቁ 114 -115 ይመልሱ | |
| 114 | የመረጃ ምንጭ ከየት ነዉ? | በረድዮ/በተለብዥን--1 ከባለሙያ----2 ከትምህርት ቤት-----3. ከጓደኛ---4.  ከዘመድ----5. ለላ ካለ ይጠቀስ-------- |  | |
| 115 | በረዲዮ/ተለበዥን ከሆነ ስንት ጊዜ? | ሁል ጊዜ -----1, በሳምንት አንድ ጊዜ----2 በሳምንት ከአንድ ጊዜ በላይ---3 |  | |
| 116 | ስለ ጤና መድህን ሰምቶ ያዉቃሉ? | አዎን ---1, አይ----2 | አዎን ከሆኔ ጥ.ቁ 117 | |
| 117 | በእሪግዝና ወቅት በ ጤና መድህን ታቅፎ/ አባል ሆኖ ነበሪ ? | አዎን----1, አባል አልነበርኩም----------2 |  | |

| - 1. **የቤተሰቡ የሃብት ሁኔታአመላካች መጠይቅ** | | | | | | |  |
| --- | --- | --- | --- | --- | --- | --- | --- |
| 118 | ቤተሰቡ እነዚህ መገልገያ ቁሳቁሶች/ መሳሪዎች አሉት | |  | | ምላሽ | | ኮድ |
| . |  | |  | | አለ | የለም |  |
| . |  | | 1. ኤልክትሪክ | | 1 | 2 |  |
| . |  | | 2. ሬዲዮ | | 1 | 2 |  |
| . |  | | 3. ቴሌቪዘዥን | | 1 | 2 |  |
| . |  | | 4. የቤት ስልክ | | 1 | 2 |  |
| . |  | | 5. ጠረጴዛ | | 1 | 2 |  |
| . |  | | 6. ወንበር | | 1 | 2 |  |
| . |  | | 7. አልጋ | | 1 | 2 |  |
| . |  | | ሌላ ካለ ይገለጽ | |  | |  |
| 119 | ከቤተሰቡ አባለት ቢያንስ አንዱ እንዚህ ንብረቶች አሉት | | 1. ሰዐት | | 1 | 2 |  |
|  |  | | 1. ሞባይል/ተንቀሳቃሽ ስልክ | | 1 | 2 |  |
|  |  | | 3. ሳይክል | | 1 | 2 |  |
|  |  | | 4. ሞተር | | 1 | 2 |  |
|  |  | | 5.  ባጃጅ | | 1 | 2 |  |
|  |  | | 6. ጋሪ | |  |  |  |
|  |  | | 7. መኪና | | 1 | 2 |  |
|  |  | | ሌላ ካለ ይገለጽ | | 1 | 2 |  |
| 120 | የቤቱ ንጣፍ ከምንድነው የተሰራው(በማየት) | | 1. ከአፈር/አሸዋ | |  | |  |
|  |  | | 2. ከጭቃ | |  | |  |
|  |  | | 3. ከእንጨት | |  | |  |
|  |  | | 4. በሴራሚክስ | |  | |  |
|  |  | | 5. በሲሚንቶ | |  | |  |
|  |  | | ሌላ ካለ ይገለጽ | | _________ | |  |
| 121 | ለመጠጥ የምትጠቀሙት ውሃ ምታገኙት ከየት ነው? | | 1. የባንባ ውሃ | | 1 | |  |
|  |  | | 2. የጉድጋድ ውሃ | | 2 | |  |
|  |  | | 3. የምንጭ ውሃ | | 3 | |  |
|  |  | | 4. የዝናብ ውሃ | | 4 | |  |
|  |  | | 5. የግፊት ውሃ | | 5 | |  |
|  |  | | 6. የኩሬ ውሃ | | 6 | |  |
|  |  | | ሌላ ካለ ይገለጽ | |  | |  |
| 122 | ምግብ ለማብሰል የምትጠቀሙት? | | 1. ኤሌክትሪክ | | 1. | |  |
|  |  | | 2. ባዮጋዝ | | 2. | |  |
|  |  | | 3. ናፍጣ/ቡታጋዝ | | 3. | |  |
|  |  | | 4. ከሰል | | 4. | |  |
|  |  | | 5. እንጨት | | 5. | |  |
|  |  | | 6. ኩበት | | 6. | |  |
| 123 | ከቤተሰቡ አባለት ውስጥ የባንክ/የኦሞ ማይክሮ ፋይናንስ ቁጠባ ደብተር ያለው አለ | | 1. አዎ  2. የለም | | 1  2 | |  |
| 124 | ቤተሰቡ ምናክል የቤት እንስሳቶች አሉት? | | 1. የወተወት ላም | |  | | |
|  |  | | 2. በሬ | |  | | |
|  |  | | 3. ላም | |  | | |
|  |  | | 4. ጊደር | |  | | |
|  |  | | 5. ዶሮ | |  | | |
|  |  | | 6. ፍየል | |  | | |
|  |  | | 7. በግ | |  | | |
|  |  | | 8. አሀያ | |  | | |
|  |  | | 9. ፈረስ | |  | | |
|  |  | | 10. በቅሎ | |  | | |
| **በባለፉት 12 ወራት የነበረው የመሬት አጠቃቀምና የሰብል ምርት ሁኔታ.** | | | | | | | |
| 125 | ቤተሰቡ የእርሻ መሬት አለው? | 1. አዎ  2. የለውም | |  | | | |
| 126 | ለእርሻ አገልግሎት የሚውል መሬት በሄክታር? | 1. --------------- | | 2. አይታወቅም | | | |
| 127 | በዚህ አመት ውስት ለእርሻ የሚውል መሬት አከራይታቹሃል? | 1. አዎ  2. አይደለም | |  | | | |
| 128 | ከባለፈው አመት ጀምሮ በዋናነት ያመረታቹት ምርት ? | 1. ጤፍ | |  | | | |
|  |  | 2. ገብስ | |  | | | |
|  |  | 3. ስንዴ | |  | | | |
|  |  | 4. በቆሎ | |  | | | |
|  |  | 5. እንሰት | |  | | | |
|  |  | 6. ቡና | |  | | | |
|  |  | 7. ጫት | |  | | | |
| 129 | የተገኘው ምርት በኪሎ? | 1. ጤፍ | |  | | | |
|  |  | 2. ገብስ | |  | | | |
|  |  | 3. ስንዴ | |  | | | |
|  |  | 4. በቆሎ | |  | | | |
|  |  | 5. ቡና | |  | | | |
| 130 | ምንያክሉ ወደ ገበያ ቀርቦ ተሸጠ? | 1. ጤፍ | |  | | | |
|  |  | 2. ገብስ | |  | | | |
|  |  | 3. ስንዴ | |  | | | |
|  |  | 4. በቆሎ | |  | | | |
|  |  | 5. ቡና | |  | | | |

| **S.no** | **ምድብ፡2 : ከወልድ ጋር የተያያዙ መጠየቆች** | **ምላሽ** | | | | | | | | **ዝለል** | |
| --- | --- | --- | --- | --- | --- | --- | --- | --- | --- | --- | --- |
| 201 | ስለ በተሰብ ምጣኔ አገልግሎት ሰምቶ ያዉቃሉ? | አዎን----1, ሰምቼ አላቅም----------2  መልሶ አዉ ከሆን ጥ፣ቁ 202 ይመልሱ | | | | | | | |  | |
| 202 | ከእርግዝና በፊት የበተሰብ ምጣኔ ተጠቅሞ ያዉቃሉ(ሁሉም አይነት)? | አዎን----1, አይ----------2 | | | | | | | |  | |
| 203 | ለመጨረሻ ጊዘ እረጉዝ ስሆኑ እድመዎት ስንት ይሆናል መገመት ይችላሉ? | ሙሉ እድመ----------1 አላቅም---------9 | | | | | | | |  | |
| 204 | የመጨረሻ ልጅሽ ስንተኛሽ ነዉ? | ------------------------- | | | | | | | |  | |
| 205 | እርግዚናሽ በምን ላይ የተመሰረተ ነበር? | በእቅድ-- ---1. ያለእቅድ-----2.  ዕቅድ ነበረኝ ግን ለወደፊት ነበረ ያቀድኩት---3 | | | | | | | |  | |
| 206 | የቅድመ ወልድ ክትትል ስንት ጊዜ አደረጉ? | 1 ግዜ-----1, 2ግዜ-----2  3 ግዜ-----3. >=4--------4 | | | | | | | |  | |
| 207 | ለመጀመርያ ጊዜ ቅድመ ወልድ ክትትል ስታደርግ እርግዚናሽ ስንት ወሩ ነበሪ? | --------------------------- | | | | | | | |  | |
| 208 | የቅድመ ወልድ አገልግሎት የት ነበረ ያደረግሽዉ? | 1. ሆስፊታል 2. ጤና ጣቢያ  3. ጤና ከላ 4. ቤት ዉስት | | | | | | | |  | |
| 209 | የቅድመ ወልድ ክትትል አገልግሎት የሰጠሸ ማነዉ? | 1. ሃክም፤.ጤና መኮንን፤ አዋላጅ ነርስ  2. የጤና ኤክስተንሽን ባለሙያ.  9. አላቅም | | | | | | | |  | |
| 210 | በቅድመ ወልድ ክትትል ግዜ የምሰጡ አገልግሎት ያዉቃሉ? | አዎን-------------1  አይ---------------2 | | | | | | | |  | |
| 211 | መልሶ አዎን ከሆነ በቅድመ ወልድ ክትትል ወቅት የተሰጤሽ አገልግሎት ምን ምን ናቸዉ? | አዎን | አይ | | | | | | |  | |
|  | የደም ግፊት መጠን ተለክቷል? |  |  | | | | | | |  | |
|  | የደም ናሙና ለምርመራ ተዎስዷል? |  |  | | | | | | |  | |
|  | የሽንት ናሙና ለምርመራ ተዎስዷል? |  |  | | | | | | |  | |
|  | የተታነስ ክትባት ሁለት ግዜና ከዛ በላይ ተሰጥቷል? |  |  | | | | | | |  | |
|  | ኤች ኣይ ቭ ምርመራ ተደርጓል? |  |  | | | | | | |  | |
|  | በ እረግዝና ግዜ ልከሰት ስለምችል አደገኛ ምልክትና በ አመጋገብ ዙርያ የጤና ትምህርት ተሰጥቷል? |  |  | | | | | | |  | |
|  | የደም ማነስ በሽታ የምከላከል እንክብል ተሰጥቷል(90+)? |  |  | | | | | | |  | |
| 212 | ለወልድ አገልግሎት ዝግጅት አድርጎ ነበረ? መልሶ አዉ ከሆነ እንደት? | አዎን---1, አይ--2 | | | | | | | |  | |
|  |  | አዎን | | አይ | | | | | |  | |
|  | የወልድ ቦታ መርጦ ነበሪ? |  | |  | | | | | |  | |
|  | የወልድ አገልግሎት ሰጭ ባለሙያ መርጦ ነበሪ? |  | |  | | | | | |  | |
|  | ለወልድ የቅርብ ጤና ተቋም መርጦ ነበሪ? |  | |  | | | | | |  | |
|  | የድንገተኛ ጊዜ ትራንስፖርት አዘጋጅቶ ነበሪ? |  | |  | | | | | |  | |
|  | ለድንገተኛ ግዜ ህክምና ገንዘብ አጠራቅሞ ነበሪ? |  | |  | | | | | |  | |
|  | በወልድና በምጥ ግዜ አብሮ የምቆይ ሰዉ መርጦ ነበሪ? |  | |  | | | | | |  | |
|  | በወልድ ግዜ እንደአሰፈላግነቱ ደም ምለግስ ሰዉ አዘጋጅቶ ነበሪ? |  | |  | | | | | |  | |
|  | በወልድ ግዜ ቤት ዉስት ያሉ ህጻናትን የምጠብቅና የምከባከብ ሰዉን አዘጋጅቶ ነበሪ? |  | |  | | | | | |  | |
| 213 | በእርግዝና ወቅት ልከሰቱ የምችሉ አደገኛ ምልክቶች መረጃ ነበሮት? | አወን----1, አይ------2 | | | | | | | |  | |
| 214 | መልሶ አዉ ከሆነ ምን ምን ናቸዉ? | የደም መፊሰስ---1,  ከፊተኛ ራስ ምታት--2,  ማነቀጥቀጥ------3,  ከፊተኛ የሆነ የሆድ ህመም---------4 | | | ከፊተኛ ትኩሳት--5  ለላ ካለ ይጠቀስ---- | | | | |  | |
| 215 | የት ወልዷል? | በጤና ተቋም----1, እቤት ዉስጥ---2 | | | | | | | |  | |
| 216 | ጤና ተቋም ከሆነ፡ በወቅቱ ምን ምን አገልግሎት አግንቶ ነበሪ ያስታዉሳሉ? |  | | | | | | | |  | |
|  | **ለ ጨቅላ ህፃን የተሰጠ አገልግሎት፡**  **ከአንድ በላይ መልስ መመለስ ይቻላል፡፡** | **ለእናት የተሰጠ አገልግሎት;**  **ከአንድ በላይ መልስ መመለስ ይቻላል፡፡** | | | | | | | |  | |
|  | 1. የህፃኑን ሰዉነት ከራስሽጋር ማገነኘትና ማሞቅ  2. የዕንበሪት እነክብካበ  3. ህፃኑ በተወለደ በአንድ ሳት ዉስጥጡት ማጥባት  4. ክትባት መስጠት  5. ክብደት መለካት  6. ለላ ካለ ይጠቀስ  7. ምንም አይነት አገልግሎት አላገኘም | 1.የደም ግፊት መጠን መለካት  2.በወልድ ግዜ ልከሰት የምችል አደገኛ ምልክት ዙሪያ ምክር መስጠት  3.ስለበተሰብ ምጣኔ ዙሪያ ምክር መስጠት  4.የበተሰብ ምጣኔ አገልግሎት መስጠት  5. ለላ ካለ ይጠቀስ----------  6.ምንም አገልግሎት አልተሰጠኝም | | | | |  | | |  | |
|  |  |  |  |  |  |  |  | | |  |  |
|  |  |  |  |  |  |  |  | | |  |  |
|  |  |  |  |  |  |  |  | | |  |  |
|  |  |  |  |  |  |  |  | | |  |  |
|  |  |  |  |  |  |  |  | |  |  |  |
|  | | | | | | | |  |  |  |  |
| 217 | በወልድ ጊዜ የባለሙያ እርዳታ አግንተዋል? | አዎን------1. አይ----------2 | | | | | | | |  | |
| 218 | \| የወልድ ሁነታ እነደት ነበረ? \| \| --- \| | በመሳርያ ያልታገዘ/ኖርማል ----1  በመሳርያ የታገዘ--------2  ቦኦፊረሽን/በቀዶጥገና---3 | | | | | | | |  | |
| 219 | በወልድ ግዜ አገልግሎት የሰጠሸ/ያዋለደሽ ምን ሙያ ያለዉ ነዉ? | ዶክተር፤ ነረስ፣አወላጅ ነረስ፣ጤና መኮንን--1  የጤና ኤክስተንሽን-----2  ባለሙያ ያልሆነ-----3  የልምድ አዋላጆች----4 | | | | | | | |  | |
| 220 | የመጨረሻ ልጅሽ ስንተኛሽ ነዉ? | 1ኛ-----1, 2ኛ-------2, 3-5ኛ-------3, >5ኛ----4 | | | | | | | |  | |
| 221 | ከወልድ በወኃላ ለምከሰቱ ቺግሮች መረጃ አሏት | 1. አዎን. 2. አይ | | | | | | | |  | |
| 222 | መልሶ አዎን ከሆኔ፡ ምን ምን ናቸዉ | 1.ከፊተኛ የሆኔ ደም መፊሰስ  2.ከፊተኛ የሆኔ ትኩሳት  3.ሽታ ያለዉ ከማህፀን የምወጣ ፈሳሽ  4.ሽንት ያለመቆጣጠሪ  5.የጡት ህመም | | | | | | | |  | |
| 223 | በጊዜዉ የድህረ ወልድ ክትትል አድርገሽ ነበረ? | 1. አዎን. 2. አይ  መልሶ አዎን ከሆነ ጥ.ቁ.222-226 ይመልሱ | | | | | | | |  | |
| 224 | መልሶ አዎን ከሆነ፡ ለምን ጉዳይ ነበረ የሄዱት | 1. እነድዉ ለመታየት ቢዬ 2. ስላመመኝ 3. ልጀን ስላመመዉ 4. ለላ ካለ ይጠቀስ | | | | | | | |  | |
| 225 | የመጀመሪያ ዙር ድህረ ወልድ ክትትል መች አደረጉ? | 1.በሁለት ቀን ዉስጥ  2.ከሶስት እሰከ ሰባት ቀን ባለዉ  3.ከሰባት ቀን እስከ ሁለት ሳምንት ባለዉ  4.ከሁለት ሳምንት እስከ ስድስት ሳምንት ባለዉ  5.አላቅም | | | | | | | |  | |
| 226 | የመጀመሪያ ዙር ድህረ ወልድ ክትትል የት አደረጉ? | 1. ሆስፊታል 2.ጤና ጣቢያr 3. ጤና ከላ  4. እቤት | | | | | | | |  | |
| 227 | ስንት ግዜ አደረጉ | አንድ ግዜ----1. ሁለት ግዜ---2. ሶስት ግዜ--3. ከሶስት ግዜ በላይ----4. አላቅም----99 | | | | | | | |  | |
| 228 | የድህረ ወልድ ክትትል አገልግሎት የሰጠሸ ማነዉ? | 1.ሃክም /ጤና መኮንን/ አዋላጅ ነርስ  2. የጤና ኤክስተንሽን ባለሙያ.  3. ለላ ካለ ይጠቀስ  99. አላቅም | | | | | | | |  | |
| 229 | በጊዜዉ ምን ምን አገልግሎት አገኘሽ  (ከ አንድ መልስ በላይ መምረጥ ይቻላል) | **የህጻናት አገልግሎትን በተመለከተ** | | | | **አዎን** | | **አይ** | |  | |
|  |  | የእንብርት ንፅና አጠባበቅ | | | |  | |  | |  |  |
|  |  | አስፈላግ ክትባት ማግኘት | | | |  | |  | |  |  |
|  |  | ክብደት መለካት | | | |  | |  | |  |  |
|  |  | በህጻናት አመጋገብ ስራት ዙርያ ት/ት መግኘት | | | |  | |  | |  |  |
|  |  | ለላካሌ ይጠቀስ--------------- | | | |  | |  | |  |  |
|  |  | መንም አይነት አገልግሎት አልተሰጠኝም | | | |  | |  | |  |  |
|  |  | **የእናቶችን አገልግሎትን በተመለከተ** | | | | **አዎን** | | **አይ** | |  |  |
|  |  | ስለ በተሰብ ምጣነ የምክር አገልግሎት ማግኘት | | | |  | |  | |  |  |
|  |  | የበተሰብ ምጣነ አገልግሎት ማግኘት | | | |  | |  | |  |  |
|  |  | ስለ አደገኛ ምልክት የምክር አገልግሎት ማግኘት | | | |  | |  | |  |  |
|  |  | የደም ማነስ መከላከያ መድሃንት መዉሰድ | | | |  | |  | |  |  |
|  |  | ለላ ካለ ይጠቀስ----------- | | | |  | |  | |  |  |
|  |  | አልተሰጠኝም | | | |  | |  | |  |  |
