# Supplementary material for: Level of completion along continuum of care for maternal and newborn health services and factors associated with it among women in Arba Minch Zuria woreda, Gamo zone, Southern Ethiopia: A community based cross-sectional study
Source: PLoS One. 2020 Jun 8;15(6):e0221670. doi: 10.1371/journal.pone.0221670 (PMC7279583; doi:10.1371/journal.pone.0221670)
Supplement: S1 File — (DOCX) [file pone.0221670.s004.docx]

**Additional definitions and measurements**

Household wealth index: a composite indicator of socio-economic status of women derived using principal component analysis based on information from housing characteristics and ownership of households’ durable goods. In this study, the factor scores of the first component were divided into quintiles.

Receiving essential elements of ANC service: Seven essential elements of ANC services were considered as the full services and these include blood pressure measurement, blood sample collection, urine sample collection, tetanus toxoid(TT2+) vaccination, iron folate(90+) supplementation, HIV testing, health education on danger signs and nutrition. Women’s receipt of the essential services was classified as “received full services” and “not received full services”. A woman was regarded as received full services if she reports that she received all the seven essential ANC services otherwise she was regarded as not received full services ((Ahmed Fathy Hamed(2018), (Choolwel Jacobs (2018)).

Exposure to mass media: Women’s exposure to mass media was measured based on their reports to whether they had the habit of watching TV or listening to radio to access relevant information on maternal and child health services. Those women who do not have such habit were considered as not having a good exposure to mass media otherwise they were regarded as having good exposure to mass media.

Membership to community-based health insurance (CBHI) was measured based on women’s report of enrollment in the CBHI in the previous year using a Yes/No option.
