# Supplementary material for: Level of completion along continuum of care for maternal and newborn health services and factors associated with it among women in Arba Minch Zuria woreda, Gamo zone, Southern Ethiopia: A community based cross-sectional study
Source: PLoS One. 2020 Jun 8;15(6):e0221670. doi: 10.1371/journal.pone.0221670 (PMC7279583; doi:10.1371/journal.pone.0221670)
Supplement: S2 File — (DOCX) [file pone.0221670.s005.docx]

**Bivariate binary logistic regression**

Table: Bivariate analysis for selection of factors associated with CoC for maternal and newborn and child health services among postnatal women in Arba Minch Demographic and Health Surveillance Site, Feb 15-March 15, 2019.

| Variables | Yes (%) | No (%) | p-value | COR(95% CI) |
| --- | --- | --- | --- | --- |
| Women’ age(n=432) |  |  |  |  |
| 18-24 | 12(12.9) | 81(87.1) |  | 1 |
| 25-29 | 17(8.6) | 180(91.4) | 0.26 | 0.64(0.29,1.39) |
| 30-34 | 9(10.2) | 79(89.8) | 0.57 | 0.77(0.30,1.91) |
| >=35 | 4(7.4) | 50(92.6) | 0.308 | 0.54(0.16,1.76) |
| Women’s education status(n=432 |  |  |  |  |
| Unable to read or write | 11(6.4) | 162(93.6) |  |  |
| Able to read or write | 6(11.8) | 45(88.2) | 0.207^*^ | 1.96(0.68,5.60) |
| Primary level of education | 17(11) | 138(89) | 0.140^*^ | 1.81(0.82,4.00) |
| Secondary and above | 8(15.1) | 45(84.9) | 0.052^*^ | 2.62(0.99,6.89) |
| Women’s employment status(n=432 |  |  |  |  |
| Non-employed | 20(7.5) | 247(92.5) |  | 1 |
| Employed | 22(13.3) | 143(86.7) | 0.049^*^ | 1.90(1.01,3.60) |
| Husband’s education status(n=412 |  |  |  |  |
| Unable to read or write | 7(6.7) | 97(93.3) |  |  |
| Able to read or write | 6(10.3) | 52(89.7) | 0.420 | 1.60(0.51,5.00) |
| Primary level of education | 18(10) | 162(90) | 0.352 | 1.54(0.62,3.82) |
| Secondary and above | 8(11.4) | 62(86.6) | 0.284 | 1.78(0.62,5.18) |
| Wealth index(n=432 |  |  |  |  |
| Lowest | 10(11.9) | 74(88.1) |  | 1 |
| Second | 10(11.8) | 75(88.2) | 0.978 | 0.98(0.38,2.51) |
| Middle | 10(11) | 81(89) | 0.849 | 0.91(0.36,2.32) |
| Fourth | 6(7) | 80(93) | 0.276 | 0.55(0.19,1.60) |
| Highest | 6(7) | 80(93) | 0.276 | 0.55(0.19,1.60) |
| Means of transport to health facilities (n=432) |  |  |  |  |
| On foot | 37(10.3) | 322(89.7) |  | 1 |
| By motorcycle/car | 5(6.8) | 68(93.2) | 0.367 | 0.64(0.24,1.69) |
| Perceived required time to reach h/facilities (n=432) |  |  |  |  |
| >= 30 minutes | 11(6.1) | 169(93.9) |  | 1 |
| < 30 minutes | 31(12.3) | 221(87.7) | 0.036^*^ | 2.155(1.05,4.41) |
| Exposure to mass media (n=432) |  |  |  |  |
| Yes | 26(10.8) | 214(89.2) | 0.385 | 1.34(0.69,2.57) |
| No | 16(8.3) | 176(91.7) |  | 1 |
| CBHI (n=432) |  |  |  |  |
| Yes | 22(9.9) | 200(90.1) | 0.892 | 1.05(0.55,1.97) |
| No | 20(9.5) | 190(90.5) |  | 1 |
| Pre-pregnancy utilization of contraception (n=432) |  |  |  |  |
| Yes | 37(12.6) | 257(87.4) | 0.006^*^ | 3.83(1.47,9.97) |
| No | 5(3.6) | 133(96.4) |  | 1 |
| Women’s autonomy on seeking maternity health care (n=432) |  |  |  |  |
| Autonomous | 35(10.2) | 309(89.8) | 0.532 | 1.31(0.56,3.06) |
| Non-autonomous | 7(8) | 81(92) |  | 1 |
| Knowledge on key pregnancy danger signs (n=432) |  |  |  |  |
| Not-knowledgeable | 18(6.9) | 243(93.1) |  | 1 |
| Knowledgeable | 24(14) | 147(86) | 0.016^*^ | 2.20(1.16.4.19) |
| Birth order |  |  |  |  |
| First | 10(13.7) | 83(86.3) |  | 1 |
| Second | 10(10.5) | 85(89.5) | 0.530 | 0.74(0.29,1.88) |
| Third | 10(8.3) | 111(91.7) | 0.232^*^ | 0.57(0.22,1.44) |
| Four and above | 12(8.4) | 131(91.6) | 0.227^*^ | 0.57(0.24,1.41) |
| Desire on pregnancy (n=432) |  |  |  |  |
| Not planned | 4(4) | 95(96) |  | 1 |
| Planned | 38(11.4) | 295(88.6) | 0.038^*^ | 3.06(1.06,8.79) |
| Time for first ANC booking (n=432) |  |  |  |  |
| At or after 16 wks. | 15(4.5) | 322(95.5) |  | 1 |
| Before 16 wks. | 27(28.4) | 68(71.6) | 0.001^*^ | 4.77(2.47,9.21) |
| BPCR (n=432) |  |  |  |  |
| Not-well prepared | 20(6.6) | 281(93.4) |  | 1 |
| Well Prepared | 22(16.8) | 109(83.2) | 0.002^*^ | 2.84(1.48,5.40) |
|  |  |  |  |  |

|  |
| --- |

Key: ^*^ indicates variables with p-values <0.25 which were candidate for multivariate logistic regression analysis

Variables with p-value less than 0.25 and candidate for multivariate logistic regression analysis were women’s education status, women’s employment status, pre-pregnancy contraception utilization, pregnancy desire, birth preparedness and complication readiness, time for ANC visits, perceived required time to reach health facilities, knowledge on key pregnancy danger signs.
